# A Bridge Too Far – Revisited: Reframing Bruer’s Neuroeducation Argument for Modern Science of Learning Practitioners

**DOI:** 10.3389/fpsyg.2016.00377

**Published:** 2016-03-16

**Authors:** Jared C. Horvath, Gregory M. Donoghue

**Affiliations:** Science of Learning Research Centre, Melbourne Graduate School of Education, University of Melbourne, MelbourneVIC, Australia

**Keywords:** neuroscience, psychology, education, translation, levels-of-organization, learning sciences

## Abstract

In *Education and the Brain: A Bridge Too Far*, John Bruer argues that, although current neuroscientific findings must filter through cognitive psychology in order to be applicable to the classroom, with increased knowledge the neuroscience/education bridge *can* someday be built. Here, we suggest that *translation* cannot be understood as a single process: rather, we demonstrate that at least four different ‘bridges’ can conceivably be built between these two fields. Following this, we demonstrate that, far from being a matter of information lack, a *prescriptive* neuroscience/education bridge (the one most relevant to Bruer’s argument) is a practical and philosophical impossibility due to incommensurability between non-adjacent compositional levels-of-organization: a limitation inherent in all sciences. After defining this concept in the context of biology, we apply this concept to the learning sciences and demonstrate why all brain research must be behaviorally translated before prescriptive educational applicability can be elucidated. We conclude by exploring examples of how explicating different forms of translation and adopting a levels-of-organization framework can be used to contextualize and beneficially guide research and practice across all learning sciences.

Interestingly, Bruer couches this argument in terms of a ‘dearth-of-information’ – suggesting the major impediment to the actualization of neuroeducation is lack of knowledge. For instance, Bruer notes “Neuroscience has discovered a great deal about neurons and synapse, but not nearly enough to guide educational practice. *Currently*, the span between brain and learning cannot support much of a load” (p. 15; emphasis ours). Similarly, Bruer states “Educational applications of brain science may come eventually, but *as of now* neuroscience has little to offer teachers in terms of informing classroom practice” (p. 4; emphasis ours). Whether he meant to convey a sense of hope or was merely trying to temper the potentially controversial nature of his argument, Bruer’s language gives the impression that, with enough knowledge, the bridge between neuroscience and educational practice is achievable.

Doubtless inspired by this language, many researchers in the learning sciences have interpreted Bruer’s article as a challenge to effectively link the brain to instructional practice (Szucs and Goswami, 2007; Willingham and Lloyd, 2007; Samuels, 2009; Antonenko et al., 2014). Unfortunately, despite decades of work, an effective neuroscience/education bridge remains a frustrating chimera – and dogged pursuit of this goal has led to an increase in the proliferation of the very ‘neuro-myths’ that Bruer was cautioning against (Alferink and Farmer-Dougan, 2010; Pasquinelli, 2012; Howard-Jones, 2014). In fact, so frustrated have some neuroscientific researchers become, that a recent argument has been put forward suggesting that failure to construct the bridge is partly due to a lack of neuroscientific literacy amongst educators (a point we will examine in more detail later in this paper: (Devonshire and Dommett, 2010; Dekker et al., 2012; Busso and Pollack, 2015).

In this article, we will re-visit Bruer’s argument: however, we will present it not as a puzzle to be solved, but as a limitation inherent in *all* fields of scientific inquiry. We will demonstrate why, practically and philosophically, attempting to prescriptively connect neuroscience and education (at least, education as it is most commonly practiced) is not only hollow, but also irrelevant. In addition, we will demonstrate that acceptance of this premise can aid in the larger goals of the learning sciences by offering a concrete and coherent framework through which to conceive of, undertake, and effectively provide prescriptive translation of research.

## Prescriptive, Conceptual, Functional, and Diagnostic Bridges

Before beginning, it is important to note that there are at least four different ‘bridges’ that can conceivably be built between neuroscience and education: prescriptive, conceptual, functional, and diagnostic. A *prescriptive* bridge attempts to specify practices to be undertaken at the educational level on the basis of evidence derived from the neurophysiologic level. In other words, prescriptive translation aims to instruct an educator and learner on *what* to do and how to do it.

A *conceptual* bridge allows for individuals to understand or conceive of phenomena at the educational level through theories generated at the neurophysiologic level. In other words, conceptual translation allows educators and learners to broaden their explanations for and interpretations of *why* certain educationally relevant practices work – however, this type of translation is silent on what said practices should or should not entail. For instance, although some educators may be inspired to use the *concept* of Hebbian-plasticity to justify the success or failure of a specific lesson, this interpretation does not impact the content, form, or efficacy of the lesson itself.

A *functional* bridge allows for phenomena at the neurophysiologic level to constrain behaviors and cognitions at the educational level. In other words, functional translation allows for alterations of brain form and/or function to expand or restrict the number and type of educationally relevant practices an educator or learner can successfully undertake – however, again, this type of translation is silent on what said practices should or should not entail. For instance, if a learner were to suffer damage to the visual cortex leading to blindness (neurophysiologic), then any learning activities would be unavoidably constrained to activities which do not rely on vision (education). Of particular importance in this example, however, is that damage to the visual cortex does not instruct the learner as to which non-visual learning activities to undertake, how to best undertake them, or how to measure their impact.

As the distinction between *prescriptive* and *functional* bridges is somewhat subtle, it may be worthwhile to expand using a specific example. Some students with attention disorders opt to use pharmaceuticals to mitigate their symptoms and improve educational performance. This performance is improved by changing activity at the neural level. At first blush, the use of a pharmaceutical may appear to be a prescriptive bridge. However, a closer examination reveals that taking a pill *constrains* an individual’s attentional networks thereby making them more receptive to learning – but this does not *engender* learning itself. Pharmaceuticals do not inform the educator or the student as to which activities to use, how to use them, or how to measure them in order to learn language, math, or geography. Accordingly, pharmaceutical intervention represents a functional, rather than a prescriptive, bridge.

Finally, a *diagnostic* bridge allows for cognitions and/or behaviors at the educational level to be backward-mapped to and correlated with phenomena extant at the neurophysiologic level. In other words, diagnostic translation aims to describe *how* a student is learning (or failing to learn) based upon individual functional brain patterns – however, once again, although this type of translation may inspire novel ideas for learning interventions, it is silent on what these interventions should entail and how they should be enacted. For instance, if a learner was to demonstrate difficulty engaging with a reading lesson (education), knowledge of his/her neuronal activation patterns during reading activities (neurophysiologic) could be utilized to potentially determine the underlying root/s of this difficulty. Of importance, however, is that this knowledge does not inform an educator or student on *what* to do to effectively improve or otherwise alter said neuronal patterns.

As might be expected, the primary form of translation most desired and expected by practicing educators is *prescriptive* (Pickering and Howard-Jones, 2007; Hook and Farah, 2013). As such, the main argument of this article centers around the prescriptive bridge only. More specifically, we will be arguing that findings at the neuroscientific level are irrelevant to and cannot be prescriptively translated to classroom behaviors.

It is important to note that the *conceptual, functional, and diagnostic* bridges are not only possible, but also already exist across all levels of the learning sciences. With regards to the conceptual bridge, educators and learners at all levels are utilizing the neuroscientific paradigm to understand and explain their current practices (Abiola and Dhindsa, 2012), even though that framework has not directly instructed them how to perform or measure the success of those practices. With regards to the functional bridge, many educators and students consume pharmaceuticals or utilize electromagnetic devices which modulate function at the neurophysiologic level (McCabe et al., 2005; Pascual-Leone et al., 2012). These tools typically expand the number of behaviorial and/or cognitive practices a person can perform, even though they do not directly instruct him/her as to which practices to perform, how to perform them, or how to measure the success of each. With regards to the diagnostic bridge, a number of individuals are utilizing knowledge of individual neurophysiology to determine the root causes of specific learning patterns, especially disabilities (Temple et al., 2001). This information may inspire learning interventions, though it does not dictate the parameters or content of said interventions.

Again, throughout this piece we will not be arguing that knowledge of neuroscience cannot influence conceptualization, function, or diagnostic ability at the educational level – rather, we will be arguing that it cannot be prescriptive at the educational level.

## Remembering Bruer’s Argument: Time and Effort

Bruer opens his article by outlining two major neurobiological arguments prevalent in the 1990s used to support the case for a neuroscience-guided education. First, he explores how the concept of early-life (0–10 years) synaptogenesis influenced the argument for *critical periods*: highly constrained time-windows during which educators can (*must*) expose students to specific experiences lest they fail to develop those skills required to demonstrate educational success (pp. 5–9). Next, he examines the concept of adolescent synaptic pruning and research demonstrating that ‘enriched’ environments can temper this pruning: an occurrence which suggests educators must enhance sensorial aspects of the learning environment lest students lose capacity to learn novel skills (pp. 9–10).

After outlining these arguments, Bruer demonstrates why each are over-extended, largely misrepresented, and do not prescriptively impact educational practice. With regards to critical periods, he reveals that the basic neuroscientific research exploring this concept has only ever been suggestive of critical periods within basic sensory and motor systems, such as vision or audition. As such, there is no evidence such periods exists (nor any theories as to why they would exist) within larger cognitive/behavioral systems required for success in educational settings, such as reading and arithmetic skills. With regards to ‘enriched’ environments, he points out that *every* environment containing a novel feature or activity can be considered ‘enriched’ and may temper synaptic pruning – regardless of the type/amount of novelty and the age of the learner (adults demonstrate a similar effect as younger students). Accordingly, though intriguing, this notion offers no concrete or applicable advice to educators beyond “don’t deprive students of novel experiences.”

Bruer continues by demonstrating that neuroscientific findings are more applicable to the field of cognitive (behavioral) psychology; and that, in turn, cognitive psychology findings are more applicable to education (pp. 11–15). For instance, Bruer argues that, though neuroscientists can demonstrate the time-course and evolution of synaptic growth and plasticity, cognitive psychologists can demonstrate the time-course and evolution of numerical competency. Though the former is doubtless reflected in the latter, only the latter can engender specific behaviors relevant and applicable to educators within the modern classroom.

Interestingly, he concludes by leaving the reader with hope; suggesting that failure to build the direct bridge between basic neuroscience and education is due only to a lack of knowledge. As outlined above, Bruer’s language implies it is only a matter of time, effort, and information before the bridge can and will be built.

## Reframing Bruer’s Argument: Incommensurable Levels-Of-Organization

The continued absence of a prescriptive neuroeducation bridge is not, as theorized by Bruer, due to a dearth of information – rather, it is due to a feature common to and accepted in all scientific fields: incommensurable levels-of-organization (Fodor, 1974, 1997; Rosenberg, 1994; Wimsatt, 1994; Rohrlich, 2004). However, these terms are notoriously ambiguous in both the philosophical and scientific literature – as such, it is important we explicate how these terms will be used in this article.

Common conceptions of *levels-of-organization* within living systems are compositional in nature (e.g., Oppenheim and Putnam, 1958; Wimsatt, 1994; Kim, 1999). More specifically, objects at each unique ‘level’ are thought to be composed of material from the preceding level. For instance, within biology, levels-of-organization typically progress accordingly:

Cell -> Tissue -> Organ -> Organism -> Population ->
Community -> Ecosphere -> Biosphere

As can be surmised, *tissues* are composed of *cells*; *organs* are composed of *tissues*; etc. Accordingly, a foundational explication of ‘levels-of-organization’ within living systems can be stated as a step-wise increase of compositional material.

An important corollary of this type of organization is *emergence*: a process whereby novel and coherent structures, patterns, and/or properties arise at ascending levels that are not exhibited within or predictable by preceding levels (for discussion: Bedeau and Humphreys, 2008). For instance, the unified size, shape, and functional coherence of an entire ant colony cannot be explained or predicted by exploring the behavior of an individual ant (Johnson, 2002). Accordingly, ‘levels-of-organization’ within living systems can be more explicitly stated as a step-wise increase of compositional material leading to emergent properties unpredictable by preceding levels.

To account for emergent properties at ascending levels, a series of unique *scientific fields* and *specialties* have been developed to explore and explain phenomena within each level – for instance, cytologists are devoted to studying cells, histologists are devoted to studying tissues, etc. However, in order to successfully undertake work, researchers at each level are necessarily required to utilize a unique set of questions, definitions, tools, and success criteria (Pavé, 2006). For example, when attempting to define the infectious disease *measles*, researchers at the cellular level may use crystallography to map proteins on the viral envelop to determine the binding site of this virus to individual epithelial cell receptors (Tahara et al., 2008), whilst researchers exploring this virus at the organ level may use post-mortem gross pathology to characterize the morphologic spread of viral driven necrosis within the lung (Kaschula et al., 1983). Similarly, whereas researchers at the tissue level may use an eyepiece micrometer to elucidate viral infolding of varied lymphoid tissues (White and Boyd, 1973), researchers at the population level may use aggregate survey and medical record data to map the spread and prevalence of this virus across a country (Santoro et al., 1984). Accordingly, ‘levels-of-organization’ within living systems can be further refined to mean a step-wise increase of compositional material leading to emergent properties unpredictable by preceding levels requiring unique language, tools, data, and success criteria to explicate.

Although, each of these above examples represents a valid way to conceive of, define, and elucidate measles, it is clear that each is unique and difficult to meaningfully correlate with the others. For this reason (amongst others), the concept of *incommensurability* was developed (Feyerabend, 1962; Kuhn, 1962). Incommensurability is perhaps best understood by laying out three basic concepts (as derived from both Feyerabend and Kuhn):

(1) Different paradigms are based upon different presumptions concerning what scientific questions are ‘valid’ and about what standards a solution needs to satisfy.
(2) The interpretation of observations is differentially influenced by the assumptions of different paradigms.
(3) The vocabulary and methods used within each paradigm are different: therefore, different paradigms cannot be compared in any meaningful manner.

Though, this theory was largely developed to explain the evolution of conceptual frameworks within specific scientific domains, replacing the word ‘paradigm’ in the above concepts with the word ‘levels’ reveals why work within each level is largely independent from work in other levels. Researchers at each level are necessarily required to utilize a unique set of questions (presumptions), definitions (vocabulary), tools (methods), and success criteria (solutions). For this reason, work at each level can be understood as incommensurable with work at other levels. However, it is worth pointing out that incommensurability is not the same as incomparability. Oftentimes, the varied languages and methodologies utilized at different levels will spring from the same coherent environmental references (e.g., germ-theory of disease) and explore the same topic (e.g., measles). Therefore, work between layers may be compared and will often prove non-contradictory (in fact, comparability gives rise to the *conceptual* bridge outlined earlier in this piece).

In summary, we refer to ‘ levels-of-organization’ in a living system as a step-wise increase of compositional material leading to emergent properties unpredictable by preceding levels requiring unique language, tools, data, and success criteria to explicate. Furthermore, adopting the traditional definition generated by Feyerabend and Kuhn (for review: Oberheim and Hoyningen-Huene, 2009), we hold that unique levels-of-organization are incommensurable in that they utilize different presumptions, vocabularies, methods, and solutions.

## Prescriptive Translation Between Incommensurable Levels-Of-Organization

The utilization of incommensurable methodologies and analyses within each level imposes limitations on prescriptive translation between levels. More specifically, in order to prescriptively translate between *adjacent levels*, one must adopt a number of assumptions. Returning to measles – it is conceivable that knowing how the virus binds to a single epithelial cell *may* be of some use to someone attempting to track cellular-membrane fusion within epithelial tissue (made up of 1000s of individual epithelial cells). However, until one is able to simultaneously measure activity within each individual cell within a larger tissue, assumptions must be made in order for the former to influence the latter: such as, that the behavior of individual cells will not drastically change amongst millions of competing cells, that the global extracellular environment of a tissue is not drastically different from that of an isolated cell, that the structural properties of a tissue will not greatly impact the prevalence or function of membrane receptors, and so on.

Certainly the translation between *non-adjacent* levels becomes increasingly precarious as the number and gravity of assumptions compound – however, more importantly, due to the emergence of unique and irreducible properties at ascending levels-of-organization unpredicted by preceding levels, translation between *non-adjacent* organizational levels is often devoid of any prescriptive utility (Atlan, 1993; Mazzocchi, 2008). For instance, researchers focused on elucidating the workings of an individual cell do not concern themselves with novel properties that emerge when 1000s of cells work in tandem to form a tissue (e.g., permeability, contractibility, mineralization, etc.). However, it is *precisely* these emergent properties that may influence researchers interested in the process by which many different tissues combine and interact to form an organ. Similarly, properties irrelevant to and unpredictable by the study of unique tissues that emerge only when exploring a complete organ (e.g., structure, function, chemical production, etc.) are *precisely* those properties that may prove influential to researchers interested in the process by which many different organs combine and interact to form an organism.

Returning once more to the example of measles, although the importance of viral binding within a single epithelial cell may be of use for someone interested in understanding how measles present within larger epithelial tissue, the prescriptive importance of this knowledge for someone interested in how measles present across the entire skin (made up of 1000s of *different* types of cells) is less clear. Continuing to higher organizational levels, the prescriptive importance of viral binding within a single cell is even less clear to someone interested in the effect of measles across the entire nervous system (organ system), the entire human body (organism), or an entire country of human bodies (population). Of prescriptive importance to researchers at each organizational level are those unique properties which emerge only at the immediately preceding level (Huneman, 2008). For instance, the researcher interested in how measles presents across the skin may be influenced by the effects and emergent properties of measles on the dozens of *tissues* that constitute the skin.

Interestingly, it is in this pattern we discover how non-adjacent levels can prescriptively interact: via translation through intermediary levels. More specifically, research at the *cellular* level may influence research at the *tissue* level (with the acceptance of certain assumptions), whilst research at the *tissue* level may influence research at the *organ* level (again, with certain assumptions), etc. As such, although attempting to build a prescriptive bridge between the cellular and organ levels may be futile, prescriptive bridges can be built over the cellular/tissue and tissue/organ gaps.

Although, this may appear obvious, it is extremely important to make this idea clear: when attempting to prescriptively connect *non-adjacent* levels-of-organization, one must always and completely traverse each intermediary level. It is only through translation of an object or concept into the novel languages, tools, methods, and data at *adjacent* levels that emergent properties can be accounted for and ideas from *non-adjacent* levels can tentatively interact.

## Levels-Of-Organization in the Learning Sciences

Once we understand that prescriptive bridges can only be meaningfully built between adjacent levels-of-organization, the reasons for the continued absence of the neuroeducation bridge become clear. Specifically, cognitive/behavioral (c/b) neuroscience is separated from education by an intermediate level-of-organization: c/b psychology (**Table 1**). As such, the behavioral properties that emerge at the c/b psychology level which are required for prescriptive translation to the educational level are non-extant in, non-predictable by, and largely irrelevant to c/b neuroscientific research. For instance, it makes little sense to talk about ‘reading’ as an isolated function within c/b neuroscience as there is no single part of the brain that ‘reads’ (Dehaene, 2009). Although, it does makes sense to discuss the function and connectivity of the mechanistic foundations of reading in the brain (e.g., auditory phonemic discrimination, visual letter recognition, semantic identification, etc.), reading as a unitary, measurable, and meaningful skill only emerges as an integrated behavioral set at the c/b psychology level (Coch, 2010). Again, this is not to say the foundations for educationally relevant skills do not exist in the brain – but, just as *speed* is not a property of any individual component of a car but emerges only with the effective integration of all components, so too do larger behaviors sets emerge and obtain meaning only following cumulative integration. This is why Bruer’s argument that brain science must pass through psychology in order to have incur relevance to education (at least, education as it is currently understood and practiced) is valid.

**Table 1:** Compositional levels-of-organization for the learning sciences.

Biological level	Domain	Relevant professions	Questions	Tools	Data	Example success criteria
Biosphere	State/Federal Governmental Policy	Ministers of State; Local Members of Parliament; Departmental Secretaries	What are the best long term strategies to fulfill the proposed educational and economic aims of state-mandated education?	Legislation; Policy; Government Funding, and Infrastructure	State/Nationwide Academic Achievement Patterns	Reduced Unemployment and Improved GDP
Ecosphere	District Policy	District Organizers; Denominational Board; Superintendents	What programs present the strongest cost/benefit ratio to meet the goals of district curricula?	District Policies; District Resources and Budget;	District-Wide Academic Achievement Scores/Rankings	Improved District Literacy Level Ranking
Community	Administration	Principals; Administrators; School Leaders	How can the timing and sequencing of courses across years and disciplines best meet the pedagogic and scheduling demands students, teachers, and parents?	General Curricula Programs; School Budget; School Policies	Year- and School-Wide Academic Achievement Scores/Rankings	Increased School Graduation Rates and Improved Tertiary Education Entrance
Population	Education (Practice)	Teachers; Tutors; Teaching Assistants	Which combination of integrated cognitions/behaviors and environments lead to the comprehension, memorization, and effective application of educationally relevant skills/information in a social environment?	Subject Curricula; Pedagogical Theories; Assessment Tools	Educationally Relevant Integrated Cognitions/Behaviors at the Group Level	Improved Individual and Social Employability, Well-being, and Economic Prosperity
Organism	Cognitive/Behavioral Psychology	Educational Psychologist; School Psychologist; Learning Psychologist; Cognitive/Behavioral Psychologist	How do isolated cognitions/behaviors combine to form an integrated ‘set’ (eg., arithmetic ability). How do these integrated behaviors develop, manifest across different scenarios, and evolve over time within an individual?	Psychometrics; Questionnaires; Cognitive Function Measures	**Integrated** Cognitions/Behaviors at the Individual Level	Elucidation of how an Individual Visually Scans and Interprets a Scene to Achieve a Pre-Established Goal
Organ	Cognitive/Behavioral Neuroscience	Cognitive Neuroscientist; Behavioral Neuroscientist	How are isolated cognitions/behaviors (eg., numerical magnitude recognition) reflected and integrated within and between neural regions?	Functional Magnetic Resonance Imaging; Scalp Electroenceph- algrophy; Transcranial Magnetic Stimulation	**Isolated** Cognitions/Behaviors and Functional Neural Networks	Localization of Primary Neural Linguistic vs. Non-Linguistic Auditory Processing Regions
Tissue	Systems Neuroscience	Systems Neuroscientist; Systems Biologist	How do neurons combine to form nuclei and how are unique nuclei organized?	Implanted Multi-Electrode Arrays; Animal Experimentation; Cell Culture	Neuronal Nuclei	Input/Output Patterns from the six Layers of the Lateral Geniculate Nuclei in response to Photostimuli
Cell	Cellular Neuroscience	Cellular Neuroscientist; Cytologist; Cellular Biologist	How do nerve cells function, communicate, and interact with unique chemical environments?	Patch-Clamps; Confocal Imaging; Laser Scanning Microscopy	Individual Neurons	Impact of Calcium Influx on Resting Action Potential Firing Rate

*Several social levels could be included *tangentially* to the educational and proceeding levels – these were omitted for the sake of clarity. Furthermore, several levels exploring the chemical and molecular activity within cellular structures could be placed beneath the cell level – these were omitted for the sake of clarity.*

For example, c/b neuroscience researchers interested in mapping language to specific areas of the brain will first *decompose* language into its many constituent parts (e.g., verb comprehension), develop highly artificial tasks meant to isolate a single constituent part (e.g., listen to a computerized word-list consisting of 100 unrelated, context-free verbs), and indirectly measure brain activity during task performance in a highly controlled environment (for review: Vigliocco et al., 2011). Although, it is possible to see how this type of work may influence researchers interested in determining how the constituent parts of language integrate to form a complete, emergent behavioral ‘language’ set at the c/b psychology level, a number of unproven assumptions must be accepted: such as that neural regions measured in isolation will behave in a similar manner when integrated with competing networks, that isolated behavioral functions will remain relatively stable when combined with secondary functions, that environmental influences will not drastically change the activation or function of specific nuclei, etc. (Chomsky, 1995).

When translating findings from c/b neuroscience directly to education, not only do the number and gravity of assumptions compound, but prescriptive utility evaporates. As noted, the decomposition of language, development of an artificial linguistic task, and indirect measurement of brain activity in a highly controlled environment are required in order to accomplish the goals of c/b neuroscientific elucidation. However, this process necessarily strips language of any meaning and ignores the influence of any competing neural and/or environmental factors: the very things one must understand in order to prescriptively influence linguistic classroom learning (Gibbons, 2002). Luckily, these factors emerge at the b/c psychology level with the recombination of isolated behaviors into behavioral sets and the elucidation of behavioral/environmental interactions.

For example, how does knowing that visually processing the letter “M” requires activation of neurons within the occipital lobe impact a lesson plan concerned with teaching students how to read? More important in this instance is the knowledge that reading ability is predicated upon (amongst other things) the effective integration of visual identification and phonemic discrimination: two larger behavioral sets understood and elucidated using paradigms developed at the c/b psychology level (Chall, 1996). Similarly, of what use is the knowledge that verb generation requires the activation of motor neurons to someone interested in helping a group of students learn how to speak French? Again, more important in this scenario is the knowledge that learning a novel language requires (amongst other things) the integration of object recognition and conceptual mapping: two larger behavioral sets understood and elucidated using paradigms developed at the c/b psychology level (Barsalou et al., 2003).

## Prescriptive Translation in the Learning Sciences

To see how this proposed framework leads to effective prescriptive translation in the learning sciences, it is perhaps best to start with a theoretical example. Above, we noted that evidence from c/b neuroscience suggests that the motor cortex demonstrates enhanced activation during verb generation (Grezes and Decety, 2001). From this, one could propose that the performance of relevant actions during novel verb generation may enhance language learning (interestingly, this practice has been utilized in classrooms since, at least, the late 1800s – but, for our purposes, we will continue as though it were novel: Gouin, 1892). At first blush, this suggestion may appear as a valid prescriptive translation: in fact, one could easily imagine the headlines heralding this pronouncement: “*Movement Activates the Brain and Improves Student Language Learning.*”

A closer examination, however, reveals that this is an idea which describes no constraints, parameters, or demonstrated efficacy. Although, motor cortical activation might suggest a link between movement and verb-learning, the specific type, form, content, and schedule of any relevant learning activities remains uncertain. Are all verbs and/or verb-tenses amenable to movement-based learning improvements? Would movement impact verb-learning in more complex and valid linguistic contexts? Would the interaction of multiple agents within a classroom setting impact movement practices and/or efficacy? Furthermore, each of these lingering questions – the back-bone for effective prescriptive utility (as each addresses the question of ‘*what to do*’) – ignores the most obvious unknown: does motor cortical activation even confer any impact on verb learning whatsoever?

Accordingly, rather than jump straight from neurophysiology to the classroom, prescriptive translation must first traverse c/b psychology, where integrated linguistic behaviors emerge and the relationship between motor cortical activation and language-learning can be systematically explored. It is here that researchers can determine how individuals come to understand verbs in context (Hamblin and Gibbs, 1999), link meaning between observed, performed, and linguistic forms of actions (Gleitman, 1990), generate novel verbs or verb-forms to shifting scenarios (Arad, 2003), etc. In other words, via c/b psychology, the constituent parts of language can be recombined to develop a more cohesive theory of how the integrated behavioral properties of language emerge whilst determining functional parameters for the influence of motor cortical activity on different stages of this process.

Although, prescriptive utility for teachers may begin to emerge at the c/b psychology level, additional work undertaken at the educational level within more ecologically-valid settings is still required to account for properties that emerge when learning takes place in an ecologically-valid classroom setting. At this stage, the impact of group dynamics (Cole, 1970), inter-personal communication (Savignon, 1991), feedback (Nicholas and Lightbown, 2001), and other factors influencing the efficacy of intentional teaching and learning of a language can be determined. It is from this work that prescriptive frameworks, protocols, and/or tools can be meaningfully developed to impact teaching and learning practices (with the understanding that variations will necessarily occur according to specific classroom environments and learning goals).

Moving to a real-world example, several researchers have recently suggested that utilizing an ‘uncertain reward’ paradigm during educational activities may improve classroom learning (Howard-Jones, 2011; Ozcelik et al., 2013). These authors report that this idea springs from work done at the c/b neuroscience level: more specifically, it has been demonstrated that reward uncertainty leads to both an increase in dopaminergic activity within the mid-brain and enhanced anticipatory focus (Critchley et al., 2001; Fiorillo et al., 2003).

As before, although an interesting idea, these neuroscientific concepts do not confer any prescriptive actions or parameters teachers or students can utilize, nor does it guarantee efficacy at the educational level. Again, one could easily the see sensational headlines for this idea (“*Uncertain Rewards Boost Dopamine and Improve Student Learning*”), but emergent properties integral to education simply have not been accounted for at this stage.

Therefore, rather than jumping straight into the classroom, these researchers next incorporated ideas derived from over a century of work done at the c/b psychological level. It was through this body of work exploring the interplay between engagement, motivation, and risk that it became clear behavior arising from reward uncertainty is highly influenced by goals and context. Accordingly, uncertain reward do not universally enhance engagement; rather, this works only when an individual determines the subjective context-relevant risk/reward ratio is beneficial (Von Neumann and Morgenstern, 1944). Furthermore, when the importance of outcome is elevated above process, uncertain rewards reduce motivation and engagement (Shen et al., 2015). Again, these parameters emerge only when larger behavioral sets (those non-extant in the brain as isolated functions) are explored. As stated by neuroscientist Wolfram Schulz, “There are no dedicated receptors for reward…[therefore] functions of reward cannot be derived entirely from physics and chemistry of input events but are based primarily on behavioral effects, and the investigation of reward functions requires behavioral theories that can conceptualize the different effects of reward on behavior” (Schultz, 2006; p. 91).

Finally, in order to account for the properties which emerge at the educational level, these researchers moved to the classroom where they conducted a 5-stage design-based study (Howard-Jones et al., 2015). As can be expected, during this process a number of issues unpredictable and irrelevant to the c/b psychology level emerged: for instance, issues of cognitive-load amongst teachers (divided between reward-schedules and teaching), asymmetric engagement between peer groups, and socially-driven emotional reactions. Through this work, the authors were able to iteratively design an engaging and effective learning tool: an excellent example of the prescriptive process engaging with and accounting for emergent properties at preceding levels of organization.

## The Curious Case of Learning Disabilities

To date, the majority of neuroscience work commonly cited as ‘successfully translated’ into the educational sphere concerns learning disabilities. More specifically, the delineation of the neural mechanisms that constitute larger behavioral sets and the ability to measure functional brain activity have allowed researchers to determine the underlying pathology behind varied forms of learning difficulties. Once the underlying source of a dysfunction is localized, relevant and effective remediation can be commenced.

Although, this process may appear to be *prescriptive* between education and neuroscience, a closer examination reveals this to be a form of *diagnostic* translation (with *prescriptive* elements between neuro and psych). More specifically, though understanding the underlying neural reasons *why* a student is unable to engage with a specific skill within a classroom may lead to effective remediation ideas, it does not generate any specific actions to be undertaken nor does it speak to the potential efficacy of those actions. Furthermore, the concept of ‘remediation’ is not the same as the concept of ‘learning.’ The goal of remediation is to eliminate any underlying block or barriers impeding a student’s ability to engage with learning tasks – however, this process is moot on the larger process of the learning-tasks themselves. For this reason, remediation activities are typically undertaken at an individual psychological (rather than a social educational) level.

As an example, hypoactivation of both frontal and left temporal brain regions relevant for phonological processing have been implicated in dysphonetic dyslexia (Temple et al., 2001). This knowledge has led several researchers to create intervention programs that may successfully correct this abnormal neural activity (though, the parameters for these programs were necessarily elucidated, tested, and established at the psychological level: Simos et al., 2002; Gaab et al., 2007). Although, this is an excellent example of prescriptive interplay between the neuropshysiologic and psychologic levels, it is important to note that remediation of phonological processing does not confer the ability to read. Once neural activity is more reflective of ‘neuro-typical’ patterns, a student with learning disabilities is simply better-positioned to engage with the process of learning to read at the educational level – remediation does not obviate the need to undertake the learning process. It is correct to argue that understanding the underlying neural processes behind reading and reading disabilities can inspire more effective learning activities (beyond remediation) – however, once this process commences, the earlier discussions of prescriptive translation become relevant.

A similar pattern can be seen in work done with dyscalculia. Recently, researchers have determined that one form of dyscalculia can be driven by abnormal development of or activity within the bi-hemispheric intraparietal sulci (Price et al., 2007) – areas of the brain linked to numerosity (Pinel et al., 2001; Castelli et al., 2006). As before, this knowledge has led to the development of intervention programs aimed at helping individuals connect quantities with the words and/or symbols that represent them (elucidated, tested, and established at the psychological level: Butterworth and Yeo, 2004; Butterworth and Laurillard, 2010). As before, these intervention programs serve to remedy an underlying issue thereby allowing a student to more effectively engage with mathematical learning activities – but it does not speak to those activities themselves. Again, it is correct to argue that understanding the mechanistic foundations of numeracy and dyscalculia can inspire more effective learning activities (beyond remediation) – however, once this process commences, the prescriptive translation framework becomes relevant.

## How does this Help Learning Science Researchers?

The largest implication of incommensurable levels-of-organization for researchers within the learning sciences is the elucidation of a clear prescriptive translational path. Over the last decade, large amounts of time and resources have been spent trying to argue for the relevance and creation of the bridge between *non-adjacent* levels; however, if we collectively acknowledge that this is practically and philosophically irrelevant (within *all* fields of science), we can re-direct this energy toward solidifying the bridges between *adjacent* levels. For instance, rather than attempting to deeply understand the vicissitudes of neuroscience and education, researchers interested in translation can focus their efforts, instead, on mastering neuroscience and psychology, or psychology and education: for it is between these adjacent levels that meaningful, prescriptive translation can take place.

In addition, researchers *within* individual levels can (and should) no longer feel pressured to prescriptively apply their work beyond adjacent levels, as this practice serves only to create overly-simplified models which may, in turn, evolve into neuro-myths. As a concrete example, many c/b neuroscientists have attempted to draw a parallel between neuroplasticity and classroom practice (Sylwester, 1986; Brown and Wheatley, 1995; Abiola and Dhindsa, 2012). More specifically, many utilize evidence of neuronal re-wiring during post-stroke rehabilitation as an argument for the link between neuroscience and learning (Murphy and Corbett, 2009; Abiola and Dhindsa, 2012). At first blush, this argument may appear strong; however, a closer examination reveals it to be devoid of any true prescriptive value. Rehabilitation consists of the continual repetition of simple behaviors with the hopes of restoring larger behavioral sets (e.g., a post-stroke victim may repeat a list of simple nouns for weeks-on-end in order to improve global linguistic function: Gresham et al., 1997). Of importance here is the return of *behavioral* function: how this function is mirrored in the brain, although interesting, is of no consequence to the larger therapeutic goals. Would we consider it a failure if, after rehabilitation, a stroke victim regained language function without demonstrating neural re-wiring? Of course not. Accordingly, the relevance of rehabilitation to education is not in neuroplasticity (neuroscience); rather, it is in the knowledge that the repetition of specific behaviors can be utilized to improve larger, more integrated behavioral sets (c/b psychology): the neural mechanisms underpinning that rehabilitation or learning is prescriptively irrelevant and uninformative to the practitioner at this higher level.

Finally, the elucidation of levels-of-organization may also prove beneficial to the organization of science of learning labs and journals. For instance, in order to guide prescriptive translation, larger mind/brain/education labs may consider organizing space so as to ensure those researchers at *adjacent* levels are in more direct contact than those at a *non-adjacent* levels. Similarly, science of learning themed journals may opt to organize articles according to the organizational levels. This practice may help researchers and practitioners at all levels quickly and easily locate relevant articles from their own and adjacent levels.

## How does this Help Educators?

Several learning science theorists have recently argued that educators require greater neuroscientific literacy and that neuroscience should be included in pre-service training (Devonshire and Dommett, 2010; Dekker et al., 2012; Busso and Pollack, 2015). Although, knowledge of the brain is certainly exciting and may inspire some teachers to develop novel concepts or theories to explain classroom behavior, the levels-of-organization framework reveals that prescriptive utility (what to do in the classroom) will never spring from this knowledge. As such, it should be made clear to educators that they need not understand the structure and function of the brain in order effectively perform their jobs. Again, this is not to say knowledge of the brain is useless in the classroom setting (as it confers opportunities for conceptual, functional, and diagnostic translation) – this is merely to say that said knowledge is *not required* to successfully perform and evolve the duties of education. For this reason, although interested educators should certainly be encouraged to learn more about the brain (potentially supported by *elective* courses offered during preservice years), non-interested educators should not feel pressured into learning information that will ultimately not directly impact the essential skills and practices required to succeed in their chosen profession.

Beyond a call for neuroscientific literacy, educators are being bombarded with programs and activities designed ‘with the brain in mind.’ Products like Luminostiy, IMPACT, Brainology, and CogMed are marketed as being brain-based, and educators interested in using them must concern themselves with issues of neural modularity and neuroplasticity. However, a closer look reveals that even these programs cannot avoid following a step-wise trajectory through the relevant levels-of-organization. For instance, CogMed may reorganize neuronal structures, but it does so only through the application and repetition of psychologically derived behavioral patterns. Of importance to educators is not whether these behavioral patterns *scale-down* to brain change, but whether they *scale-up* to more general, educationally relevant behavioral sets and outcomes. As such, educators need not worry about claims made regarding the ‘brain-based’ nature of a program; rather, they need only concern themselves with claims made regarding behavioral activities and educationally transferable outcomes of a program.

Again, some theorists argue enhanced neuroscience literacy amongst educators will help inoculate teachers against these types of programs (Goswami, 2006; Howard-Jones, 2014) – however, this remedy is short-sighted and misses the ultimate point. ‘Brain-based’ learning is not the first trend to sweep through education, nor will it be the last; as such, insisting teachers are proficient in the knowledge required to assess the scientific veracity of claims made by product designers will be a Sisyphean task. For instance, there is already discussion of a ‘gene-based’ education (Asbury and Plomin, 2014): does this mean teachers must next become literate in genetics? How about when personalized tutoring-systems emerge: much teachers then learn Artificial Intelligence algorithms? Rather that asking already overly-busy teachers to obtain *more* knowledge, the levels-of-organization framework clarifies that teachers need only obtain the *right* knowledge. In these instances, educational trends can be effectively and successfully addressed by ensuring teachers focus only on the evidence that matters most to their practice: more specifically, how does a program impact the learning of students within my particular educational setting? This inquiry makes obsolete any facts that do not take into account the emergent properties relevant to education (e.g., facts about the brain function, genetic coding, computational scripts, etc.). Furthermore, ensuring teachers focus their inquiries on practically *relevant* information may inspire the designers of learning products to obtain and deliver data more meaningful for their target audience.

Taking this concept further, it is conceivable that within the next decade engineers will develop a portable interface capable of measuring and analyzing brain activity on the fly (indeed, many companies already advertise prototypes of such devices). Even if one were to use this interface to develop an educational program that adapts to a student’s unique neural patterns, this would be as hollow as current brain-based programs. As before, in this instance, brain activity is *not* guiding education – it is merely guiding *sets of behaviors* that may scale-up to relevant educational outcomes. Again, the efficacy of this program would not be measured in brain-waves or brain change; rather, it would be measured in the transfer of the skill/s obtained via repetition of behaviors to the larger behavioral goals of education. Explicit knowledge of brain function and change does not obviate the need for proper competence, comprehension, and transfer as elucidated using behavioral measures. This becomes especially clear when one remembers that functional neural markers obtained at the c/b neuroscience level are necessarily reliant on the deconstruction of behaviors into constituent parts devoid of integrative meaning. Until such a time as information and skills can be directly uploaded into a student’s brain (at which point, education as we currently know it will likely cease to exist) neuroscientific measures must always pass through the intermediary *behavioral* level in order to be prescriptively relevant to education.

## A Bridge to the Future

Since Bruer first put forth his neuroeducation argument, there has been tremendous debate amongst researchers and practitioners concerning the merits of translating brain knowledge for classroom use. However, until now, very little has been done to clearly define what is meant by *translation*. This uncertainty, no doubt, has fueled this debate and obfuscated attempts to determine what can and can not be meaningfully expected from a union between neuroscience and education.

Through our explication of four different types of translation, it is clear that neuroscience and education are already linked via conceptual, function, and diagnostic bridges. Through, these forms of translation, dialog amongst educators is evolving, understanding of the holistic learning process is emerging, and options to measure and modulate neurophysiology within both typical and dysfunctional learners is expanding. However, it is also clear that neuroscience cannot prescribe practices within education except via psychology as this is the only means by which to elucidate and account for emergent properties at ascending levels of organization.

Once it is accepted that the prescriptive neuroscience/education bridge is a chimera, all the time, resources, and energy spent on trying to actualize it can be re-distributed to more fruitful prescriptive translational avenues: specifically, to the c/b neuroscience : c/b psychology and c/b psychology : education junctions. Within this framework, any theoretical over-extension can easily be identified and qualified prior to the emergence of new neuro-myths. In addition, much pressure will be removed from educators with regards to what concepts they are expected to understand and utilize within the classroom: specifically, any claim beyond behavioral enactment and measurement is irrelevant.

As with any novel scientific endeavor, it is important to establish a fundamental framework so as to beneficially guide development and application into the future. We believe the delineation of different forms of translation and explication of the brain/behavior/classroom bridge required for effective prescriptive translation provides the strongest support as we continue to advance our work and will, ultimately, lead to faster and more successful prescriptive translation between the laboratory and the classroom.

## Author Contributions

Both JH and GD conceptualized, produced, and edited this article.

## Conflict of Interest Statement

The authors declare that the research was conducted in the absence of any commercial or financial relationships that could be construed as a potential conflict of interest.
